# Sex-specific associations of serum selenium and selenoprotein P with type 2 diabetes mellitus and hypertension in the Berlin Aging Study II

**DOI:** 10.1016/j.redox.2023.102823

**Published:** 2023-07-23

**Authors:** Kamil Demircan, Sandra Hybsier, Thilo Samson Chillon, Valentin Max Vetter, Eddy Rijntjes, Ilja Demuth, Lutz Schomburg

**Affiliations:** a Institute for Experimental Endocrinology, Charité-Universitätsmedizin Berlin, Corporate Member of Freie Universität Berlin and Humboldt-Universität zu Berlin, Max Rubner Center (MRC) for Cardiovascular Metabolic Renal Research, D-10115, Berlin, Germany; b Department of Endocrinology and Metabolic Diseases (including Division of Lipid Metabolism), Biology of Aging Working Group, Charité-Universitätsmedizin Berlin, Corporate Member of Freie Universität Berlin and Humboldt-Universität zu Berlin, And Berlin Institute of Health (BIH), Berlin, Germany

**Keywords:** Berlin aging study II, SELENOP, Glucose, Insulin, Trace elements

## Abstract

**Background:**

Selenium is essential for expression and proper function of a set of redox active selenoproteins implicated in aging-relevant diseases, e.g. type 2 diabetes mellitus (T2D) and hypertension. However, data in cohorts of older adults, particularly with respect to different Se biomarkers and sex-specific analyses are sparse.

**Objective:**

To assess associations of serum Se and selenoprotein P (SELENOP) concentrations with T2D and hypertension in a cohort of older females and males.

**Methods:**

This study included 1500 participants from the Berlin Aging Study II. Diagnosis of T2D was made in case of antidiabetic medication, self-reported T2D, or laboratory parameters. Diagnosis of hypertension was based on self-report, blood pressure measurement, or anti-hypertensive medication. Se was measured by spectroscopy, and SELENOP by ELISA. Multiple adjusted regression models quantified dose-dependent associations.

**Results:**

Participants had a median(IQR) age of 68 (65,71) years, and 767 (51%) were women. 191 (13%) participants had T2D and 1126 (75%) had hypertension. Se and SELENOP correlated significantly (r = 0.59, p < 0.001), and were elevated in those with self-reported Se supplementation. Serum Se and SELENOP were not associated with T2D in the whole cohort. In men, SELENOP was positively associated with T2D, OR (95%CI) for one mg/L increase in SELENOP was 1.22 (1.00,1.48). Se was non-linearly associated with hypertension, comparing to the lowest quartile (Q1), and participants with higher Se levels (Q3) had a lower OR (95%CI) of 0.66 (0.45,0.96), which was specific for men. SELENOP positively associated with hypertension, and OR (95%CI) per one mg/L increase was 1.15 (1.01,1.32).

**Conclusions:**

The data suggest a sex-specific interrelationship of Se status with T2D and hypertension, with apparent biomarker-specific associations.

## Highlights

• This study included 1500 senior subjects from the Berlin Aging Study II.
• Selenium displayed a U-shaped association with hypertension in men.
• SELENOP dose-dependently associated with hypertension in women.
• SELENOP dose-dependently associated with T2D in men.

## Graphical abstract

**Figure:**
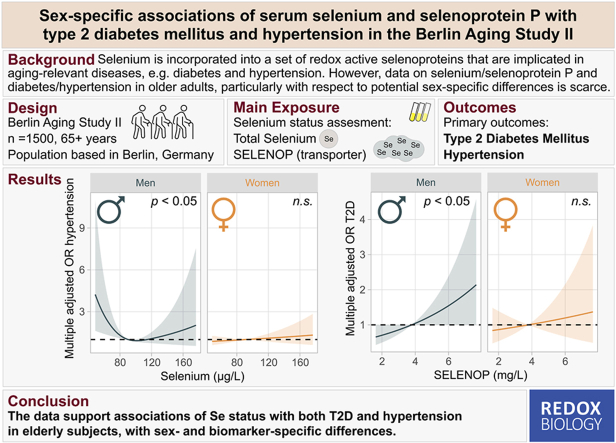

## 1 Introduction

Type 2 diabetes mellitus (T2D) and hypertension are among the most common diseases among older adults, together contributing strongly to the global mortality burden [1,2]. The risk factors leading to T2D and hypertension are interrelated [3,4]. Among currently known modifiable risk factors, obesity and diet play an important role for both [5]. While composition of diet such as type (e.g. Western or Mediterranean) or composition of macronutrients have been identified as relevant risk factors [[6], [7], [8]], the role of micronutrients is poorly understood.

Among those, selenium (Se) is an essential micronutrient incorporated into selenoproteins in form of selenocysteine, hereby facilitating important physiological functions such as ROS prevention, redox signalling and thyroid hormone metabolism [9,10]. Liver-derived selenoprotein P (SELENOP) is the main serum protein involved in Se transport to peripheral tissue [11], where Se is needed for the biosynthesis of other selenoproteins, e.g. glutathione peroxidases, deiodinases, or thioredoxin reductases [12]. Beside transport, SELENOP also has enzymatic activity [13,14]. Mechanistic studies have linked Se and SELENOP to insulin resistance and pulmonary arterial hypertension [15,16]. Despite this line of evidence, clinical data for SELENOP is sparse. Most observational studies on Se status and T2D or hypertension have quantified total Se from serum or plasma only, which constitutes a compound parameter including several selenocompounds of which SELENOP is the most abundant biomarker indicating systemic Se transport [17]. Moreover, the potential relevance of sex-specific differences have rarely been taken into account [[18], [19], [20]]. Accordingly, the database is currently incomplete, both with regard to the association of Se and SELENOP status with T2D and hypertension risk and course, and potential differences between men and women [21,22].

In order to address this knowledge gap, we investigated the association of serum Se and SELENOP concentrations with T2D and hypertension in the population-based Berlin Aging Study II (BASE-II) cohort of elderly community-dwelling individuals.

## 2 Methods

### 2.1 Study population

This study included 1500 participants of the BASE-II study. BASE-II is a population-based study of community-dwelling elderly subjects situated in the greater area of Berlin, Germany. Study design of BASE-II has been described earlier in detail [[23], [24], [25]]. All included participants gave written informed consent to participate in the study, which was carried out in accordance with the Declaration of Helsinki. Ethical approval for the study was provided by the ethics committee of Charité - University Medicine Berlin (approval number: # EA2/029/09).

### 2.2 Outcomes and covariates

T2D and hypertension were assessed as main outcomes. T2D was determined according to guidelines of the American Diabetes Association (ADA), i.e., either fasting plasma glucose ≥126 mg/dl, glucose ≥200 mg/dl 2 h after oral glucose tolerance testing (OGTT), or HbA1c ≥ 6.5%, use of antidiabetic medication, or self-reported T2D [26]. Hypertension was determined according to 2018 ESC/ESH guidelines, i.e., either blood pressure ≥140/90 mmHg, use of antihypertensive medication, or self-reported hypertension. Seated blood pressure was assessed as mean between measurements of the left and right arm. When only one measurement was available, the singular value was used. Information on medication was retrieved in one-to-one interviews or from a medication list. Information on smoking and alcohol intake were retrieved from the medical history by a study physician. Weight, height, BMI and waist-to-hip ratio were measured with the electronic measuring station seca763 (SECA, Germany) [23].

### 2.3 Assessment of selenium and selenoprotein P

Detailed information on blood sampling was described earlier [23]. Serum samples were stored in −80 °C freezers until shipment to the laboratory for analysis of Se biomarkers at the Institute of Experimental Endocrinology, Charité Berlin, by scientists and technicians blinded to clinical data. Total reflection X-ray fluorescence (TXRF) spectroscopy was used to determine total Se status using a TXRF spectrometer (Bruker Nano GmbH, Berlin, Germany), as described in detail earlier [27,28]. Participants’ serum were diluted 1:2 with a buffer containing 1000 μg/L Gallium to serve as standard, and 8 μL of the dilution was applied on quartz glass slides provided by the manufacturer (Bruker Nano GmbH) for measurement. Serum SELENOP concentrations were measured with a sandwich ELISA method using monoclonal antibodies targeting human SELENOP, as described [20]. The ELISA was calibrated against a standard reference material (SRM1950).

### 2.4 Statistical analysis

Clinical characteristics of study participants are presented as median along with interquartile range (IQR) for continuous variables, and as frequency (%) for categorical variables. Interdependence of Se biomarkers was assessed applying Spearman's rank correlation.

In regression models, predictor variables were entered as continuous variables or quartiles in case of non-linearity. Associations of Se biomarkers with T2D or hypertension were assessed applying linear regression aided by restricted cubic splines (RCS) with three knots (0.1, 0.5, 0.9) to allow for non-linearity. Applying likelihood ratio test, RCS models were compared to linear models, and p for non-linearity <0.05 was considered as non-linear. Models were adjusted for age (years), BMI (kg/m^2^), waist-to-hip ratio (continuous), smoking status (current, cessation <1 year ago, cessation ≥1 year ago, never) and alcohol intake (never, ≤1/month, 2–4/month, 2–3/week, ≥4/week). An interaction term between sex and Se biomarker was implemented into the models and p_interaction_<0.15 was considered a significant interaction.

Distribution of missing information was checked visually, and found to be missing at random. Accordingly, missing variables were imputed using multiple imputation by chained equations [29], whereas predictive mean matching, logistic regression, and polytomous regression were used to impute continuous, binary categorical, and unordered categorical variables, respectively [30]. Imputed variables, outcome and predictor variables were included in the imputation model. Ten imputations with ten iterations were performed. Convergence of the model was assessed, main analyses were compared to complete case analyses, to check for robustness.

All analyses were conducted in R, on the RStudio environment using the packages dplyr, tidyr, ggplot2, rms, hmisc, and mice.

## 3 Results

### 3.1 Study population and characteristics

A total of 1500 participants with a median (IQR) age of 68.0 (65.0, 71.0), of whom 767 (51%) were women, were included in the final analyses. 191 (13%) had T2D, and 1126 (75%) had hypertension. Further anthropometric, clinical and lifestyle characteristics of the study population are displayed in Table 1.

**Table 1:** Characteristics of study participants.

Characteristic	N = 1500^a^
**Age (years)**	68.0 (65.0, 71.0)
**Women**	767 (51%)
**BMI (kg/m**^**2**^**)**	26.3 (23.9, 29.0)
**Waist-to-hip ratio**	0.96 (0.90, 1.02)
**T2D**	191 (13%)
**Fasting glucose (mg/dl)**	92 (86, 101)
**2h-OGTT**^b^**(mg/dl)**	104 (86, 124)
Missing	179
**HbA1c (%)**	5.50 (5.30, 5.80)
**Hypertension**	1126 (75%)
**Systolic RR (mmHg)**	141 (130, 153)
**Diastolic RR (mmHg)**	83 (76, 90)
**Smoking**	
Current	140 (9.4%)
Cessation <1 year ago	13 (0.9%)
Cessation ≥1 year ago	631 (42%)
Never	709 (47%)
**Alcohol**	
Never	23 (1.7%)
≤ 1/month	260 (19%)
2–4/month	337 (25%)
2–3/week	329 (24%)
≥ 4/week	416 (30%)
Missing	135
**Selenium (μg/L)**	91 (78, 104)
**Selenoprotein P (mg/L)**	3.79 (3.16, 4.41)
**Selenium supplementation**	95 (6.4%)

Missing not shown if <2%.

a Median (IQR); n (%).

b OGTT was only assessed in participants that were not already diagnosed with T2D before examination.

### 3.2 Correlation of Se biomarkers and effects of supplemental Se intake

**Fig. 1:**
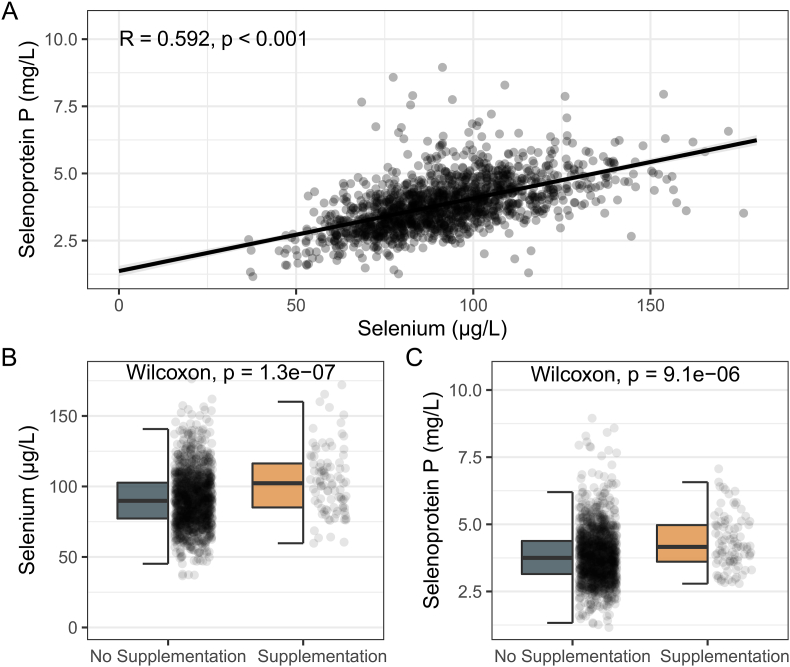
**Correlation of selenium biomarkers and effects of supplemental Se intake. A** Spearman's correlation of serum selenium and selenoprotein P in the whole cohort. **B** Selenium concentrations according to self-reported supplementation. **C** Selenoprotein P concentrations according to self-reported selenium supplementation. Thick solid line of half boxplots depicts median, upper end of the box depicts the upper and lower end depicts the lower quartile, upper and lower lines depict whiskers.

Se and SELENOP concentrations were significantly correlated across the whole cohort (Spearman's R = 0.592, p < 0.001) (Fig. 1A) and effect sizes were similar in separate subgroup analyses of women (Spearman's R = 0.589, p < 0.001) and men (Spearman's R = 0.595, p < 0.001). Se concentrations were significantly higher in participants with self-reported Se supplementation (Fig. 1B). Similarly, SELENOP levels were higher in those with self-reported Se supplementation (Fig. 1C).

### 3.3 Association of Se and SELENOP with T2D and hypertension

**Fig. 2:**
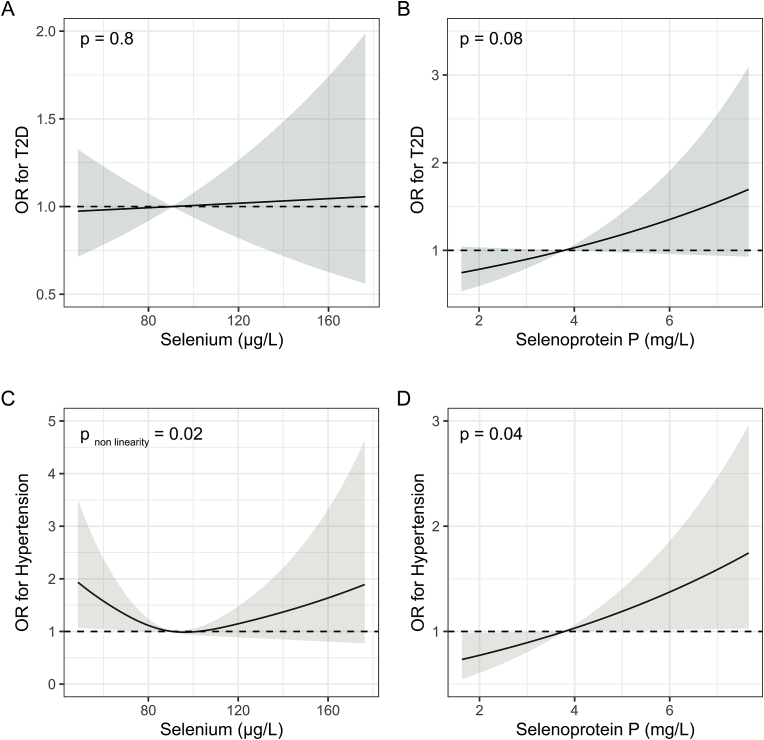
**Multiple adjusted restricted cubic spline regression analyses. A** Association of selenium with T2D. **B** Association of selenoprotein P with T2D. **C** Association of selenium with hypertension. **D** Association of selenoprotein P with hypertension. Shaded areas depict 95% confidence intervals.

In the main analyses, participants with self-reported Se supplementation were excluded, as it may not reflect the long-term Se status of the individual and bears the risk of contributing to reverse causation of the associations. Se was not associated with T2D in univariate or fully adjusted analyses (Table 2, Fig. 2A). There was a trend for a positive linear association of SELENOP with T2D in univariate (p = 0.079) and fully adjusted (p = 0.083) analyses (Table 2, Fig. 2B). Se was non-linearly (p_non-linearity_ = 0.022) associated with hypertension in a U-shaped manner (Table 2, Fig. 2C). When comparing to the lowest quartile (Q1), participants in the third quartile of Se (Q3) had a lower OR for hypertension in fully adjusted analyses (OR (95%CI) = 0.66 (0.45, 0.96), p = 0.031). SELENOP was linearly positively associated with hypertension in univariate (p = 0.023) and fully adjusted (p = 0.039) models (Table 2, Fig. 2D).

**Table 2:** Regression analyses displaying associations of Se biomarkers with T2D and hypertension.

		Univariate^a^			Fully Adjusted^b^		
	n	OR	95% CI	p-value	OR	95% CI	p-value
**T2D**							
Selenium^c^	1378	1.00	0.99, 1.01	0.7	1.00	0.99, 1.01	0.8
SELENOP^c^	1378	1.14	0.98, 1.33	0.079	1.15	0.98, 1.34	0.083
**Hypertension**							
Selenium							
Q1	345	–	–		–	–	
Q2	345	0.72	0.50, 1.04	0.080	0.78	0.53, 1.14	0.2
Q3	344	0.57	0.40, 0.81	**0.002**	0.66	0.45, 0.96	**0.031**
Q4	344	0.70	0.48, 1.00	0.052	0.78	0.53, 1.15	0.2
SELENOP^c^	1378	1.16	1.02, 1.32	**0.023**	1.15	1.01, 1.32	**0.039**

^**†**^ Non-linear.

a Non-adjusted univariate analyses.

b Adjusted for age, sex, BMI, waist-hip-ratio, smoking status and alcohol intake.

c Continuous variable, OR reported as per one unit increase.

### 3.4 Sex-specific association of Se and SELENOP with T2D and hypertension

**Fig. 3:**
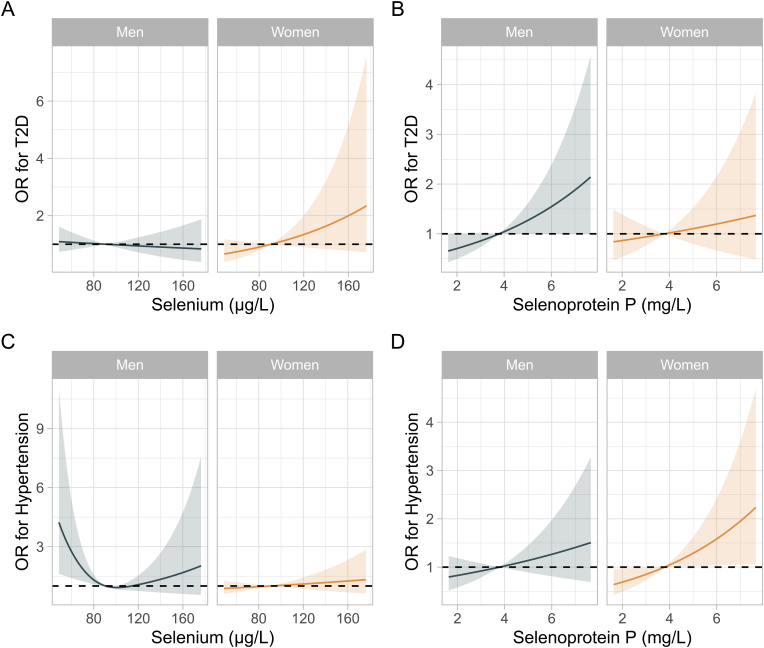
**Sex specific multiple adjusted restricted cubic spline regression analyses. A** Sex-specific association of selenium with T2DM. **B** Sex-specific association of selenoprotein P with T2D. **C** Sex-specific association of selenium with hypertension. **D** Sex-specific association of selenoprotein P with hypertension. Shaded areas depict 95% confidence intervals.

There was no association of Se concentrations with T2D in any sex-specific subgroup (Table 3, Fig. 3A). In men, SELENOP was positively associated with T2D in fully adjusted models, although the interaction with sex was not significant (Table 3, Fig. 3B). The U-shaped association of Se with hypertension observed in the whole cohort was significant in the subgroup of men only (OR(95%CI) for Q3 vs. Q1 = 0.43 (0.23,0.79), p = 0.007), while no association was noted in women (Table 3, Fig. 3C). There was a significant interaction of Se with sex when considering hypertension as an outcome. SELENOP was positively associated with hypertension, however, only in women, with no significant interaction of SELENOP and sex observed (Table 3, Fig. 3D).

**Table 3:** Regression analyses displaying associations of Se biomarkers with T2D and hypertension according to sex.

		Women^a^				Men^a^			
	n	OR	95% CI	p-value	n	OR	95% CI	p-value	p _interaction_
**T2D**									
Selenium^c^	697	1.01	1.00, 1.02	0.2	678	1.00	0.99, 1.01	0.7	0.2
SELENOP^c^	697	1.08	0.83, 1.41	0.6	678	1.22	1.00, 1.48	**0.048**	0.5
**Hypertension**									
Selenium^b^									
Q1	175	–	–		169	–	–		
Q2	184	0.90	0.54, 1.48	0.7	160	0.61	0.32, 1.15	0.13	0.3
Q3	170	0.91	0.55, 1.49	0.7	173	0.43	0.23, 0.79	**0.007**	**0.067**
Q4	168	0.98	0.59, 1.64	>0.9	176	0.56	0.30, 1.05	0.072	**0.15**
SELENOP^c^	697	1.23	1.02, 1.49	**0.034**		1.11	0.91, 1.36	0.3	0.4

a Adjusted for age, BMI, waist-hip-ratio, smoking status and alcohol intake.

b Non-linear.

c Continuous variable, OR reported as per one unit increase.

## 4 Discussion

In the BASE-II study of community-dwelling elderly subjects, there was a positive association of SELENOP with hypertension, and a U-shaped association of Se with hypertension, along with a positive trend for an association of SELENOP with T2D, which was restricted to men. Sex-specific analyses revealed a significant interaction between Se and sex. On the one hand, the U-shaped association of serum Se concentration with hypertension was limited to men. On the other hand, the positive association of SELENOP with hypertension was restricted to women.

Research on the potential role of Se status with T2D has so far been conflicting, both with regard to pre-clinical and epidemiological studies [22,31]. In mechanistic studies, elevated SELENOP has been shown to increase insulin resistance, by acting on pancreatic β cells and downregulating adenosine monophosphate-activated protein kinase (AMPK) [16,32]. Targeting SELENOP with neutralizing monoclonal antibodies was shown to ameliorate this effect [33]. In contrast, mice fed with a low Se diet containing less than 0.10 mg Se/kg exhibited dysregulated glucose metabolism and insulin sensitivity [34]. Similarly, mice receiving Se in form of selenomethionine had lower glucose concentrations along with a higher sensitivity to insulin than control mice or those receiving sodium selenite [35].

Clinically, data on different Se status biomarkers is sparse, but some epidemiological studies have shown a positive association of serum Se or SELENOP with T2D [36,37]. However, a systematic review has also reported U-shaped associations [38], and when measuring toenail Se, even inverse associations were observed [39,40]. Notably, very low Se status was associated with hypoglycaemia [41]. In the current study there was an overall trend for a positive association of SELENOP with T2D in men, in agreement with the mechanistic studies for SELENOP and most of the observational human studies [22]. In line with the evidence of an inverse association of Se status with gestational diabetes [42], there was no association observed in women.

The database for Se and hypertension is less well investigated, particularly with regard to serum SELENOP, as most studies investigated total Se only [21,[43], [44], [45], [46]]. Nawrot et al. demonstrated a higher risk of high blood pressure with low blood Se levels, which was specific to men [43,47]. This study also found the association to be exclusive to the subgroup of men, while there was no association in the subgroup of women. Surprisingly, however, there was a positive association of SELENOP with hypertension, which in turn was not seen in men, emphasizing the additional prognostic/diagnostic value of this Se status biomarker. Collectively, the data from this cross-sectional study support considerable associations of Se status with both T2D and hypertension in elderly subjects residing in an area with marginal Se supply, with sex-specific differences and biomarker-specific results.

Among the strengths of this study is the large sample size, and the access to a database with various covariates with low numbers of missing data, which enabled the identification of an independent association of the two Se biomarkers with the outcomes. The option to considering multiple aspects for diagnosis of the outcomes, including laboratory measurements, self-reports and medication use, allowed for ensuring a low misclassification bias. The assessment of two different complementary biomarkers for Se status, along with their coherent correlation and positive response to supplemental Se intake, ensured a reliable quantification of the main exposure, the Se status.

Our study has some limitations. Due to the observational, cross-sectional design, we cannot account for residual confounding or reverse causality. An important source of reverse causality is self-supplementation, as those with disease may be more likely to supplement. However, data about Se supplementation was available from questionnaires, and we were able to exclude those participants in the analyses. Serum Se and SELENOP levels were higher in subjects who supplemented, which further ensured correctness of self-reports and analyses. Lastly, the participants enrolled in the BASE-II study are in general healthier when comparing to nationwide data.

## Funding

The research has been supported by the 10.13039/501100001659Deutsche Forschungsgemeinschaft (DFG), Research Unit FOR-2558 “TraceAge” (Scho 849/6–2), and CRC/TR 296 “Local control of TH action” (LocoTact, P17). This article uses data from the Berlin Aging Study II (BASE-II) which was supported by the German 10.13039/501100002347Federal Ministry of Education and Research (BMBF) under grant numbers #01UW0808; #16SV5536K, #16SV5537, #16SV5538, #16SV5837, #01GL1716A and #01GL1716B. The funding sources had no involvement in the study design; collection, analysis, and interpretation of data; writing of the report; or the decision to submit the article for publication.

## Author contributions

KD: conceptualization, methodology, software, formal analysis, investigation, data curation, writing - original draft, and visualization. SH: investigation, methodology, writing – review & editing. TSC: methodology, software, writing – review & editing. VMV: data curation, software, writing – review & editing. ER: data curation, methodology, writing – review & editing. ID: conceptualization, investigation, resources, supervision, project administration, funding acquisition, writing – original draft. LS: conceptualization, investigation, resources, supervision, project administration, funding acquisition, writing – original draft.

## Declaration of competing interest

LS holds shares of selenOmed GmbH, a company involved in Se status assessment; no other relationships or activities that could appear to have influenced the submitted work are indicated.

## Data Availability

Due to concerns for participant privacy, data are available only upon reasonable request. Code for statistical analyses is available upon request from the corresponding authors.
